# Discrimination of Acoustic Stimuli and Maintenance of Graded Alarm Call Structure in Captive Meerkats

**DOI:** 10.3390/ani11113064

**Published:** 2021-10-27

**Authors:** Sebastian Schneider, Sarah Goettlich, Charlette Diercks, Paul Wilhelm Dierkes

**Affiliations:** Department of Bioscience Education and Zoo Biology, Goethe-University Frankfurt, Max-von-Laue-Str. 13, 60438 Frankfurt, Germany; sarah.goettlich@onlinehome.de (S.G.); charlette.diercks@gmail.com (C.D.); dierkes@bio.uni-frankfurt.de (P.W.D.)

**Keywords:** meerkats, suricates, bioacoustics, graded structure, vocalization, alarm calls, playback experiment, fuzzy clustering, LASSO algorithm, zoo, natural behavior, animal welfare, acoustic features

## Abstract

**Simple Summary:**

Preserving natural behaviors has many advantages for both research and animal welfare. Natural behaviors include producing vocalizations and responding to them. If it can be shown that the natural vocal repertoire is preserved in zoos, studies in zoos may help to expand the knowledge of acoustic behaviors and transfer it to animals in the wild. Once the meaning of diverse vocalizations is known, inferences can be made about an animal’s internal state in order to adapt and improve conditions for animals in zoos. In this paper, a natural and selective response of meerkats to potentially threatening acoustic signals such as the call of a predator is demonstrated. It can be shown that both the graded structure of meerkat alarm calls, which serves to convey the urgency of a dangerous situation, and the natural response to alarm calls are preserved. The obtained findings allow a continuation of the bioacoustic studies known for wild meerkats in zoos. The meerkat’s ability to already recognize acoustic signals as a potential threat may be crucial information for certain husbandry conditions. Vocalizing predators kept or naturally occurring near the meerkat enclosure form one example. The level of stress induced by potential threats and the associated alertness could be determined by using the graded alarm calls as a tool.

**Abstract:**

Animals living in human care for several generations face the risk of losing natural behaviors, which can lead to reduced animal welfare. The goal of this study is to demonstrate that meerkats (*Suricata suricatta*) living in zoos can assess potential danger and respond naturally based on acoustic signals only. This includes that the graded information of urgency in alarm calls as well as a response to those alarm calls is retained in captivity. To test the response to acoustic signals with different threat potential, meerkats were played calls of various animals differing in size and threat (e.g., robin, raven, buzzard, jackal) while their behavior was observed. The emitted alarm calls were recorded and examined for their graded structure on the one hand and played back to them on the other hand by means of a playback experiment to see whether the animals react to their own alarm calls even in the absence of danger. A fuzzy clustering algorithm was used to analyze and classify the alarm calls. Subsequently, the features that best described the graded structure were isolated using the LASSO algorithm and compared to features already known from wild meerkats. The results show that the graded structure is maintained in captivity and can be described by features such as noise and duration. The animals respond to new threats and can distinguish animal calls that are dangerous to them from those that are not, indicating the preservation of natural cooperative behavior. In addition, the playback experiments show that the meerkats respond to their own alarm calls with vigilance and escape behavior. The findings can be used to draw conclusions about the intensity of alertness in captive meerkats and to adapt husbandry conditions to appropriate welfare.

## 1. Introduction

When it comes to animal welfare in zoos, the main topics studied include enrichment, social conditions, and enclosure design [1]. Among other things, the aim is to ensure that animals have a natural frequency and range of behaviors and to take action to expand the existing behavioral repertoire to that end [2]. Behaviors natural to animals in the wild should be preserved in captivity for a number of reasons. The individual animals are healthier and more active, protecting them from physical health problems and maintaining a strong immune response [3]. Maintaining good health plays a role in high reproductive success [4], which is necessary for captive breeding programs to keep a healthy population. Furthermore, stress-free animals increase the visitors’ satisfaction [5,6], their knowledge of natural, i.e., adaptive, animal behavior, and thus their interest in animal welfare and biodiversity conservation.

Acoustic communication plays an important role in natural behavior, as sender and/or receiver can benefit from the meaning of the information contained in acoustic signals [7]. The information conveyed by acoustic signals from animals ranges from species identification, as in frogs and insects [8], to individual identification and personal traits, as in gibbons [9,10], banded mongoose [11], giant otters [12,13], or dolphins [14]; to contextual information such as resource availability, as in chimpanzee food calls [15]; or predator threat, as in meerkats [16], marmots [17], or primates [18,19]. Accordingly, a natural vocal repertoire in captive animals can provide extensive information about the physiological state, sex, subspecies, reproductive state, social status, stress, and animal welfare [20]. This information can provide the foundation for improving animal welfare and lead to a better understanding of animal biology.

In addition to factors that promote welfare, there can also be factors that can reduce welfare. Stress is a key factor here, causing animals to suffer disease or fail to reproduce or develop properly [21]. It has already been shown that even the calls of predators are sufficient to reduce reproduction or endanger the rearing of offspring through fear-induced reduction in food supply [22,23,24]. This can become a problem in zoos if vocalizing predators are kept within auditory range or occur naturally in the environment. Again, preservation of the natural repertoire in zoos can be beneficial, as alarm calls triggered by predators can be used to monitor stress for animals that feel threatened [25].

However, proving that certain species communicate naturally in captivity and that parts of the vocal repertoire have not been lost over generations is challenging. In many cases, those vocal parts are unlearned sounds that have not been used or triggered for a long time due to the absence of an appropriate trigger in zoos. To verify that the acoustic response of the animals occurs naturally, conditions in which the corresponding vocalization is normally emitted can be simulated. The occurrence or absence of acoustic responses then provides information about the degree of loss or preservation of the corresponding vocalizations. Analysis and comparison of vocalizations occurring in captivity with those occurring in the wild poses another problem. In bioacoustics, vocalizations are usually described using extracted features of the time domain and frequency spectrum [26]. However, since there is no uniform system for this feature analysis, it can be difficult for researchers from different institutions to prove whether the sounds recorded in the zoo correspond to those in the wild. This can be especially problematic when dealing with vocalizations that are very similar to each other, or graded vocalizations where features change fluidly depending on the situation or their state of arousal. In the case of graded vocalizations, a major challenge for the observer is to objectively assess how the animals evaluate the degree of gradation in a given situation. Therefore, an additional, computer-based assessment is useful. Clustering methods such as fuzzy clustering are able to classify the graded structure of vocalizations [27]. If it can subsequently be shown that the features used by the clustering algorithm to distinguish the different degrees of gradation match the features used to describe gradation in the wild, this can be seen as evidence for the preservation of graded vocalizations. One way to determine which features best describe the differences in gradation degrees is the LASSO algorithm [28].

The alarm calls of meerkats (*Suricata suricatta*) are an example of graded vocalizations. Meerkats in the wild are known to change features such as noise, duration of vocalization, and dominant formants with increasing urgency for aerial, terrestrial, and recruitment alarm calls [16]. However, an appropriate use of these calls apparently has to be learned by young animals [29]. It has also been shown in other mammalian species that the amount of exposure to calls influences learning in young animals [30,31,32,33,34]. This suggests that the graded structure of alarm calls may be unlearned in the continued absence of danger, resulting in captive meerkats being unable to discriminate between urgency. In addition, young meerkats need experience during their development to properly associate an alarm call with the type of threat and the appropriate response [34]. In the wild, meerkats have been shown to respond appropriately to alarm calls played back, even in the absence of a predator [35]. This response to an alarm call could be lost over several generations if alarm calls occur too infrequently or not at all. Meerkats in captivity have been shown to respond to olfactory [36] and visual stimuli with adequate alarm calls [37]. However, the question arises whether meerkats can also distinguish potentially dangerous animal calls from non-dangerous animal calls using purely auditory stimuli. Furthermore, it is not known whether the graded structure of alarm calls is also maintained in zoo-dwelling meerkats and whether an adequate response to alarm calls is maintained even when no danger is imminent.

This paper aims to demonstrate that the alarm calls, which typically occur when danger is present, are graded just as they are in the wild and represent the urgency of the danger. If this is the case, the frequency and intensity of the alarm calls could be used to draw conclusions about the stress level and welfare of the meerkats. For this reason, acoustic experiments were conducted with different animal sounds, which on the one hand should trigger alarm calls of the sentinel and on the other hand should lead to an adequate behavioral response. In addition, it will be tested whether captive meerkats are able to distinguish animal calls that may pose a threat to them, from those that are harmless. Playback experiments were then conducted using the recorded meerkat alarm calls and recording the behavioral response. A subsequent feature and cluster analysis should provide information on whether the urgency level is distinguishable with this method.

## 2. Materials and Methods

### 2.1. Study Site

Experiments took place at the Opel Zoo in Kronberg, Germany, at the meerkat enclosure during summer 2017 and summer 2018. In summer 2017, three male and two female adults along with two female juveniles resided in the enclosure. In summer 2018, the same adults remained in the enclosure, with two new male and two new female juveniles.

The enclosure for the meerkats group is built on natural ground and encloses 55 m^2^. Animals can dig freely for 1.8 m before encountering a digging barrier. The enclosure barrier consists of a 1.2 m high glass and brick wall with an electrified fence. The center of the enclosure consists of two rocks with one heat lamp. Multiple rocks and branches are scattered throughout. An indoor enclosure can be reached through a rock fissure, where water, food, and shelter are provided.

### 2.2. Experimental Background

**Figure 1:**
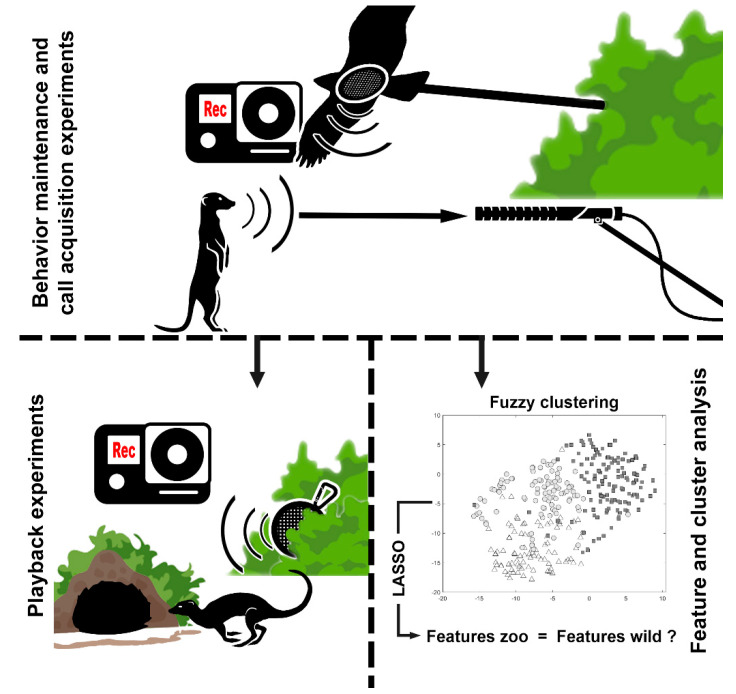
Visual representation of the three parts of this study. In the first step (experiments on behavior maintenance and call acquisition), we test whether meerkats exhibit natural behavior to detect and avoid enemies. For the following steps, alarm calls triggered by predator stimuli are recorded. The playback experiment is designed to determine whether captive meerkats still learn to respond appropriately to their own alarm calls. This is conducted by playing back the previously recorded aerial alarm calls and observing the response of the meerkats. The third approach is to investigate whether the graded structure of alarm calls is maintained in captivity. For this purpose, the acoustic features of the previously recorded aerial and terrestrial alarm calls are extracted and clustered. The LASSO algorithm is used to select the features that best describe the differences of the resulting clusters. If these features correspond to the features already described for wild meerkats, it can be assumed that the graded structure is preserved.

The experiments performed in this paper can be divided into three main parts (Figure 1).

The first step was to examine the natural protective behavior of meerkats born and raised in captivity to the simulated presence of a potential threat. We investigated how the group responded to acoustic stimuli alone and in combination with a visual stimulus that simulated an aerial predator. These experiments additionally served for the acquisition of alarm calls by the meerkats on which the playback experiments were performed later. In addition, terrestrial alarm calls were provoked by leading a dog past the edge of the enclosure. This experiments are termed “behavior maintenance and call acquisition experiments”. Emitted calls were analyzed for usability and taken for both playback experiments and cluster and feature analysis. After video evaluation, results were analyzed for heightened alert behavior. Table 1 provides an overview of the three parts of the study.

#### 2.2.1. Behavior Maintenance and Call Acquisition Experiments

In the auditory experimental study, the recording lasted two minutes before an auditory or visual stimulus and five minutes after the stimulus. The stimulus itself lasted 30 s. Two GoPro Hero 4 Silver cameras (GoPro Inc., San Mateo, CA, USA) were placed at the top of the enclosure to record the whole enclosure during the seven-minute period. The video recordings were evaluated with the freeware Boris [38]. This allowed us to continuously observe each individual during the seven-minute period. Eight animal vocalizations were downloaded from the “Tierstimmenarchiv Museum für Naturkunde Berlin” (www.tierstimmenarchiv.de, accessed on 30 July 2016) and used as acoustic stimuli. These included two local birds of prey, the common buzzard and the red kite; two small song birds, robin and blue tit; two middling-sized birds, the cuckoo and the common raven; as well as one bird of prey local to the meerkats’ natural habitat, the jackal buzzard. Additionally, we used a call of a mammal predator, the common jackal, that is neither local to the meerkats in situ nor to their ex situ habitat as a new potential predator. The recordings were played via a mobile phone connected to a JBL Clip 2 Bluetooth speaker (JBL, Los Angeles, CA, USA). For the visual stimulus, a silhouette of a common buzzard was cut out from cardboard. The proportions were customized to achieve a wing span of 1.2 m, equivalent to that of a common buzzard in the wild. An imprinted plastic foil was added to the dummy bird for greater authenticity. The dummy was mounted on a three-meter-long telescopic pole and moved above the meerkats’ enclosure. The person who moved the buzzard dummy lay at the ground hiding near bushes for one minute before starting the recording period of seven minutes and remained there still until the recording period finished, to be sure not to influence the meerkats’ behavior. The JBL Clip 2 Bluetooth speaker was attached on top of the buzzard dummy, invisible for the meerkats, to present the buzzard vocalization in combination with the visual stimulus.

To provoke terrestrial alarm calls, a large Weimaraner dog was tempted by food to run along the border of the meerkat enclosure. No direct interaction between dog and meerkats took place but the dog was visible for the meerkats. The scenario was conducted three times. The behavior of the meerkats during the experiment to provoke terrestrial alarm calls was not evaluated, as it only served to record the corresponding terrestrial alarm calls. The sentinel alarm calls were recorded by using a t.bone EM 9900 shotgun microphone (Thomann, Burgebrach, Germany) and a Tascam DR-680MKII field recorder (TEAC, Tama, Tokio, Japan). The sampling rate was set to 48 kHz.

To avoid habituation effects, the intervals between the same stimuli were as long as possible, lasting at least three days. The aerial alarm calls, which were used for the cluster analysis, were provoked almost 14 months after performing the acoustic stimuli. For this purpose, only the buzzard dummy was used, as it always emitted alarm calls with decreasing urgency the longer the stimulus lasted. Contextual assignment of urgency was intentionally omitted in order not to impute perceived urgency to the meerkats. Thus, a human rating bias could be avoided.

#### 2.2.2. Playback Experiments

Ten days of playback experiments were conducted with aerial alarm calls recorded during behavioral maintenance and call acquisition experiments. Each day included one test resulting in 10 playback experiments. To validate normal vigilance behavior, two minutes were recorded before each call was played. After stimuli onset, a measurement period of another five minutes followed. This was used to analyze possible carry over effects on vigilance behavior from the call. To reduce learned behavioral responses, the position of the loudspeaker was varied during the experiments.

Playback experiments should be performed using synthetic stimuli to reduce pseudo-replication effects [39]. Therefore, an individual sequence was generated with randomly selected units at random intervals for each run of the playback experiment. These sequences were matched to the structure of the call sequences recorded in the behavior maintenance and call acquisition experiments. Measured vocal reaction to aerial predators included firstly 4 to 6 aerial alarm calls in rapid succession, followed by a pause and several aerial alarm calls with longer intervals. Inter-unit intervals were drawn from the unit population and restricted to the calculated standard deviation range of the mean pause length (0.89 s). This was performed to exclude outliers that lead to long breaks. Averaging across trials, the mean number of aerial alarm calls was 70. Each synthesized aerial stimuli contained 4–6 aerial alarm calls in rapid succession followed by a silent period with a length in the standard deviation range of the mean pause length. After the silent period, 70 aerial alarm calls were added. This resulted in a duration of 40–45 s per stimuli. To rule out a response to loudspeaker play only, robin sounds were played as a control sound on 10 specific days when no trial was conducted.

#### 2.2.3. Feature and Cluster Analysis

MathWorks MATLAB 2020a (The MathWorks Inc., Natick, MA, USA) was used for feature extraction, cluster analysis, and evaluation using LASSO. To describe the characteristics of the vocalizations, 23 features of the time domain and frequency spectrum were extracted for each vocalization (Table S1). The idea of the method presented here is to group the features of the recorded aerial (*n* = 312) and terrestrial (*n* = 91) alarm calls first by means of fuzzy clustering into the already known three gradation levels. If the three resulting clusters are well distinguishable by the known features such as the proportion of noise, the frequency of the formants, and the duration [16], these features should be selected in an evaluation using the LASSO algorithm.

Here, a fuzzy c-means cluster algorithm is used, where data points are not sharply delimited from each other, but each data point is assigned a certain degree of membership to a cluster [27]. This makes it possible to better cluster data that have smooth transitions instead of distinct boundaries, as is the case with graded vocalizations. The used fuzzy clustering algorithm uses the maximum number of clusters to be determined as well as the degree of “fuzziness” (µ) as parameters. The parameter µ controls the amount of fuzzy overlaps of clusters. By iteratively increasing µ, the centroids of the clusters converge. If the Euclidean distance of the centroids falls below a threshold, the according clusters merge into one. The number of clusters that persists the longest is considered the most stable and best cluster solution. For this work, a maximum number of clusters of 3 for each alarm call was chosen, as already described for wild meerkats [16]. The resulting labels are used to select the features that best describe the differences in the clusters using the LASSO algorithm [28]. LASSO belongs to the shrinkage methods by which less relevant features automatically become smaller and thus less significant. Irrelevant variables can also become equal to zero, whereby a variable selection is performed.

### 2.3. Behavior Analysis

Behavior during the behavior maintenance and call acquisition experiments as well as during the playback experiments was analyzed based on video recordings. The software BORIS v.7.7.4 (Behavioral Observation Research Interactive Software) [38] was used for continuous sampling of one focal animal at a time for the whole measurement period. To compare the different points in time, the whole observation was divided into 15 s intervals, where t0 corresponds to the interval in which the corresponding stimulus was started. Statistics were performed using SPSS Statistics v.26 (IBM, Armonk, NY, USA). Non parametrical tests were used, since a Shapiro–Wilk test yielded that the data were not normally distributed.

Behaviors such as “eat”, “foraging”, “rest”, or other behaviors such as interactions with meerkats or grooming were summarized as normally seen and inattentive behavior and named “self- and intraspecies-directed behavior”. The vigilant behaviors such as “guard” and “observe” were named “environment-directed behavior”. This results in three main behaviors for the analysis: self- and intraspecies-directed behavior, environment-directed behavior, and flight. Additionally, during the behavior maintenance and call acquisition experiments, the behaviors “walk” and “not visible” were recorded but not included in the analysis. The ethogram used is provided in Table 2. Definitions of the behaviors are provided in the Supplemental Material (Table S2). For statistics, a Wilcoxon test was used to compare both self- and intraspecies-directed behavior and the environment-directed behavior before and after the start of the stimulus. We took the mean of the eight intervals before the stimulus started and compared it with each interval after the stimulus onset. A Wilcoxon test was used to compare the flight behavior during the first 15 s interval after the stimulus onset of the different calls. The flight reactions were tested against the flight reaction during the blue tit call, where flight was never observed. All data were statistically tested with SPSS.

## 3. Results

### 3.1. Behavior Maintenance on Acoustic Stimuli

**Figure 2:**
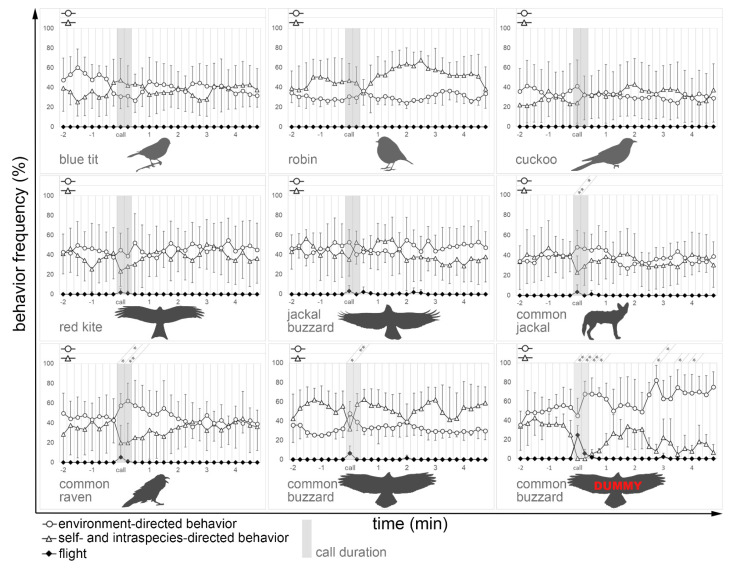
The figure shows how the self- and intraspecies-directed, the environment-directed, and the flight behavior evolve for the different stimuli. The time axis is divided into 15 s intervals. Each interval shows how frequently the associated behavior was observed (in %). The gray shaded area marks the period in which the stimulus was presented. If present, a significant difference between one interval and the mean of the eight intervals before stimulus onset for self- and intraspecies-directed behavior and environment-directed behavior can be taken from the upper part of each graph. Only significant differences are shown: (*) *p* ≤ 0.05; (**) *p* ≤ 0.01.

The calls of the common buzzard, the common raven, and the common jackal showed the greatest change in self- and intraspecies-directed behavior and environment-directed behavior, i.e., vigilance, shortly after the onset of the stimulus compared to behavior two minutes before the onset of the stimulus (Figure 2). A significantly increased level of vigilance was maintained about 15 to 30 s after stimulus onset for the common raven, the common buzzard, and the common jackal, whereas the common buzzard call showed the highest intensity of vigilance reaction (*p* < 0.01). Although the red kite and the jackal buzzard are birds of prey as well, the vigilance behavior of the meerkats was not significantly increased after stimulus onset. Significant decreases were found for self- and intraspecies-directed behavior, i.e., inattention, for a duration of 15 to 30 s when the calls of the common raven, the common buzzard, and the common jackal were played. Among them, the raven and jackal calls showed the highest intensity of decrease (*p* < 0.01), while the buzzard call led to lower (*p* < 0.05) and shorter duration of decreased inattention (Figure 2). The small local bird calls, i.e., blue tit and robin, as well as the cuckoo as a middle-sized bird, showed no significant changes in behavior after presenting one of the auditory stimuli neither in vigilance nor in inattentive behavior or flight.

When using the buzzard dummy in combination with the common buzzard call, there is a strong decrease in self- and intraspecies-directed behavior. A significant decrease in self- and intraspecies-directed behavior maintains about 1 min and 15 s after stimulus onset. There is also another significant decrease in self- and intraspecies-directed behavior from the third to the fourth minute after stimulus onset (Figure 2).

The presented stimuli elicited appropriate behavioral responses and, in most cases, alarm calling. Feature extraction revealed a mean fundamental frequency (F0) of 250 Hz (standard deviation: ±65 Hz) for the recorded aerial alarm calls. This corresponds to the range of 200–300 Hz reported in the literature for most harmonic calls of meerkats [40]. The flight response decreased from the paired stimulus condition (*p* < 0.001) to the auditory presentation of the buzzard call (*p* < 0.01), right down to the jackal (*p* < 0.05) and the jackal buzzard (*p* < 0.05). Although vigilance behavior increased significantly during the raven call, no significant increase in flight behavior could be determined. Small song bird calls as well as the cuckoo call and the call of a red kite had no significant effects on their vigilance and flight response nor on their self- and intraspecies-directed behavior. In addition, the Wilcoxon test showed a significant difference between the paired stimulus condition of the dummy bird and buzzard call compared to the common buzzard call alone (*p* < 0.05).

### 3.2. Playback Experiments

**Figure 3:**
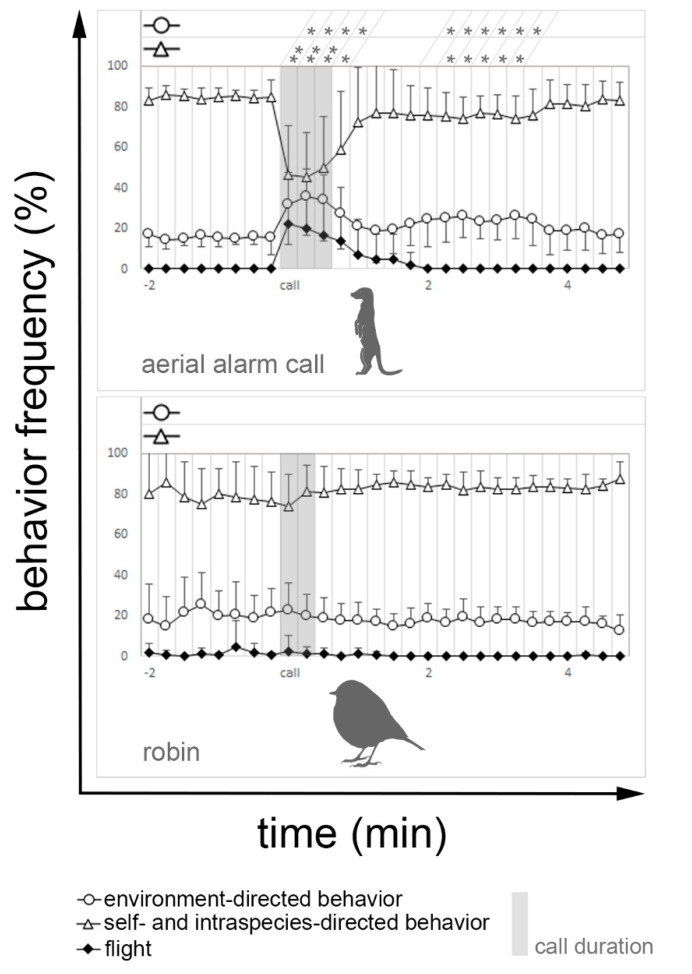
The figure shows how the self- and intraspecies-directed, the environment-directed, and the flight behavior evolve for the playback of the aerial alarm call and the control stimuli (robin). The time axis is divided into 15 s intervals. Each interval shows how frequent the associated behavior was observed (in %). The gray shaded area marks the period in which the stimulus was presented. If present, a significant difference between one interval and the mean of the eight intervals before stimulus onset for self- and intraspecies-directed behavior and environment-directed behavior can be taken from the top of each diagram. Only significant differences are shown: (*) *p* ≤ 0.05; (**) *p* ≤ 0.01.

Meerkats respond to their own aerial alarm calls with significant changes in all three behavioral categories (Figure 3). Environment-directed behavior showed significantly increased vigilance even after the end of the stimulus (*p* < 0.05). The self- and intraspecies-directed behavior was significantly decreased during the stimulus (*p* < 0.01) and one interval beyond (*p* < 0.05). Furthermore, for both environment-directed and self- and intraspecies-directed behavior, a significant change in behavior can again be determined for slightly more than one minute after the onset of the stimulus. Flight was also significantly increased over four 15-second intervals (*p* < 0.05). When playback of robin calls was used as a control, no significant changes in behavior were detected.

### 3.3. Feature and Cluster Analysis

**Figure 4:**
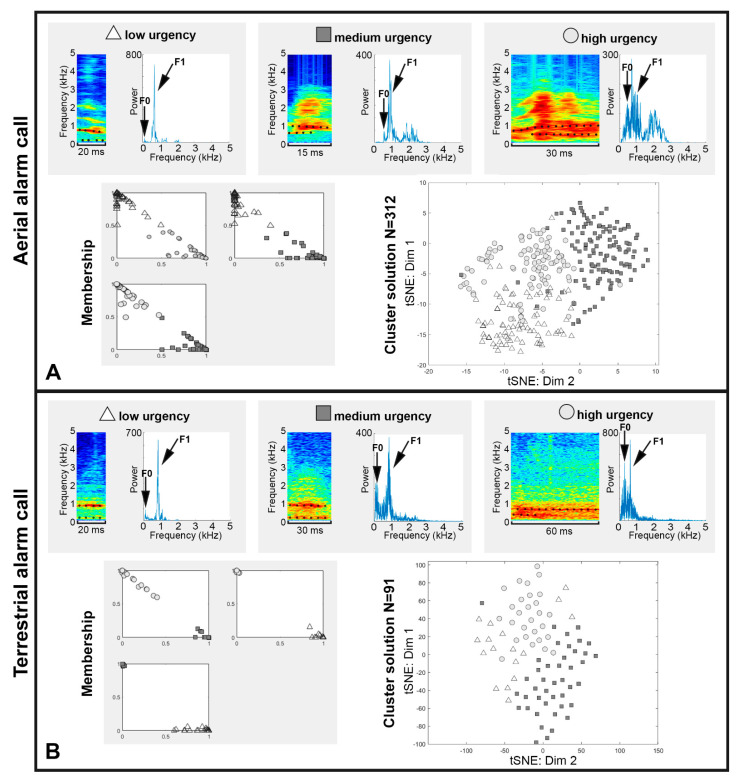
Fuzzy clustering results for both alarm call types ((**A**) = aerial alarm calls; (**B**) = terrestrial alarm calls), with one example per urgency level (triangle, square, and circle). For each urgency level, the features duration, F0 and F1, are indicated. Each vocalization is shown as both a spectrogram (left) and a spectrum (right). In both, the noise component within the vocalizations is well discernible via the broad, non-specific distribution of the high-energy frequencies. The diagram “Membership” provides a pairwise comparison of cluster segregation. Each call is assigned a membership value between 0 and 1 for the associated cluster, where 1 corresponds to a 100% membership. Each diagram compares two urgency clusters (triangles, squares, and circles indicate which urgency cluster the individual data points belong to). In this way, the membership values (x- and y-axes) can be used to determine the affiliation of the individual calls to the respective urgency cluster and to estimate the degree of convergence between the urgency clusters.

The aerial alarm calls recorded during the call acquisition experiment via the buzzard dummy and terrestrial alarm calls provoked by a dog were used to examine the alarm calls of the meerkats for a graded structure. Cluster analysis using fuzzy clustering resulted in the maximum number of 3 clusters for both alarm call types (Figure 4). Based on the resulting labels, the LASSO algorithm was used to determine the corresponding features for each alarm call type that best described the differences in the vocalizations of the different clusters (Table 3). Figure 4 shows the three identified urgency levels for each alarm call type and provides an overview of the most important features.

The acoustic features identified by the LASSO algorithm show that a change in the characteristics of the vocalizations is best described by the duration, as well as the dominant frequency bands (F0, F1, DFA1maloc, DFA2maloc) and the noise component (harmonic ratio, spectral flatness). The clustering of vocalizations represented by t-SNE in two dimensions (Figure 4) also shows that there do not seem to be sharp boundaries between urgency levels. Accordingly, the results confirm a graded structure of the alarm calls, which can be described by features such as the duration, the dominant frequency bands, and the noise component.

## 4. Discussion

### 4.1. Meerkats Can Discriminate Acoustic Stimuli in Terms of Their Potential Significance

Zoo animals may lose the ability to recognize their natural predators if they reside in zoos for several generations, making reintroduction programs potentially less successful. For example, captive-born collared peccary (*Pecari tajacu*) has been shown to fail in discriminating between predator and non-predator models [41]. Reduced anti-predator behavior may also occur in the wild, depending on suitable environmental conditions. For example, isolation on islands, where species occur in an environment with few or no predators, may result in the actual loss or alteration of anti-predator behavior [42]. Our data show that captive-born meerkats exhibit natural anti-predator behavior in response to acoustic stimuli, with potential predators inducing stronger responses. However, the strongest increase in vigilance and flight behavior was achieved by using the buzzard dummy as visual stimulus in combination with the auditory buzzard call. This was to be expected, as it can be assumed that the perceived intensity is enhanced by the combination of visual stimuli with auditory stimuli [43]. Based on the significant response to the common raven and the common buzzard but not to the red kite, we conclude that the meerkat group may have learned from previous experiences with those animals and is able to categorize their potential thread. In addition, the meerkats responded significantly with increased vigilance and flight to predator calls that were unfamiliar to them, such as the common jackal call. Small songbirds as well as the cuckoo call did not result in a significant increase in vigilance behavior or flight response. Therefore, a response based only on the playback by means of the loudspeaker should be excluded. In summary, meerkats show graded responses to calls, with potential threats leading to higher vigilance or flight. This finding of graded behavioral responses is consistent with the already known responses to different olfactory stimuli in captive meerkats [36,37]. In the experiments, the behavioral responses were mostly of short duration. No significant changes were observed shortly after the acoustic stimulus was discontinued. Prolongation of this duration occurs only when a visual stimulus is presented in conjunction with a bird of prey call. This coupled stimulus seems to sensitize the animals to potential threats, as they increase their vigilance behavior and significantly decrease their self- and intraspecies-directed behavior from time to time. Although there was no additional stimulus, this continued for several minutes after the onset of the stimulus itself. This indicates an overall normal cooperative behavioral repertoire, as non-guarding individuals rely on their sentinel and quickly resume their normal behavior, but remain in a more vigilant state when confronted with a greater threat.

The playback experiments indicate that captive meerkats naturally respond to their own alarm calls, even when there is no immediate threat. This suggests that individuals understand the specific call and maintain an appropriate response to alarm calls. Because an appropriate response to alarm calls must be learned in meerkats, the retention of this response could mean that meerkats perceive various situations as threatening and emit alarm calls even in the “safe” environment of a zoo. Since there is evidence of teaching for meerkats [44], it would be conceivable that even in the absence of danger, the young are taught an adequate response to alarm calls.

### 4.2. Feature and Cluster Analysis

Comparing vocal repertoires between studies conducted by different people is difficult, because judgments and decisions made on weighting different features in a pattern can differ between individuals. This makes evaluations difficult to quantify because individuals are usually unaware of the thresholds they are using [45]. For this reason, the classification in this study was performed using appropriate algorithms. The results of the feature and cluster analysis show that a graded structure of alarm calls is also maintained in captive meerkats to indicate the urgency of a threat. This gradation is best distinguished by the features duration and noise, which have been demonstrated both in the wild [16] and in the present study in captive meerkats. While formants could also be identified as potential features to distinguish vocalizations, these could also indicate different individuals emitting the sound. This hypothesis is supported by a study which could show interindividual variations of the formant pattern in meerkats alarm calls [46]. Assuming that the urgency of the alarm calls is subject to the motivation-structural rules, where aggressive vocalizations become noisier in general [47], it is possible that the graded structure does not need to be learned and is thus preserved over several generations.

### 4.3. Relevance for Animal Welfare and Conservation

The complete preservation of the vocal repertoire and the associated natural response in zoo-habituated meerkats has implications for research and animal welfare. It enables studies to be conducted in zoos under facilitated conditions, allowing the results to be transferred to animals in the wild. In addition, it is possible to build on the studies already known and continue the research in zoos. The graded structure makes it possible to estimate the degree of alarm status in order to draw conclusions about the stress level. Thus, husbandry conditions where a high rate of alarm calls with high urgency occur can be adjusted in favor of the animals. This may be particularly relevant for groups living in mixed species enclosures or enclosures close to predators, as well as for enclosures with a high occurrence of birds of prey. In this context, the results obtained here show that a potential predator does not necessarily have to be within sight, but that calls alone are sufficient to trigger increased vigilance and flight behavior. Thus, stressful situations can constantly arise in a “safe” environment, which can have a negative impact on the animal welfare as well as on the reproduction and offspring rearing [22,23,24]. The vocal behavior of captive animals can be used as an indicator of their well-being. For captive brown capuchins (*Cebus apella*), for example, it was shown that terrestrial predator alarm vocalizations are a valid monitor of stress [25]. In this regard, it is interesting to determine across many species whether alarm calls can be observed in captivity or whether the ability to produce these calls is reduced in captive animals, at least in some species. Within primates, Campbell monkeys (*Cercopithecus campbelli*) are an example of captive animals having a limited alarm call repertoire compared to wild animals. Possible reasons include limited exposure to predators in general or during infant development [48]. Potential changes in alarm communication in captivity may be particularly relevant to reintroduction projects. Morris et al. 2021 address in depth the maintenance of captive behavior, particularly the maintenance of alarm communication. They developed a list of recommendations and actions to improve the alarm communication before and during an relocation process [49]. This is intended to help increase the likelihood of successful release.

### 4.4. Limitations

Despite the advantages of computer-aided evaluation over human evaluation, it is not guaranteed that the cluster solution obtained also reliably reflects reality. In particular, in the case of graded vocalizations, where discrete boundaries cannot be drawn between clusters, it is difficult to assign vocalizations whose features can be ranked between two clusters. In order to obtain a cluster solution that is as reliable as possible, the typicality coefficient and the silhouette value were determined. Using these values, the parameters of the fuzzy clustering algorithm can be set in the best possible way. The typicality coefficient determines the difference of the two largest membership values for each vocalization [27]. The larger this value is, the more clearly the corresponding vocalization can be assigned to a particular cluster. By iteratively changing the parameters, the cluster solution at which these values are highest can be found. Based on the silhouette value, the best labeling could additionally be determined for each iteration. Since the most stable number of clusters was further used, a robust and reliable cluster solution can be assumed.

Since the data used here were only collected in one zoo, the results cannot be strictly applied to meerkat groups in other zoos. At Opel Zoo Kronberg, visitors are allowed to have dogs on a leash. This could create situations with different levels of threat for the meerkats. However, during the observation period, no significant reaction to visitors’ dogs could be detected. Provoking terrestrial alarm calls by means of a dog was performed on the side of the enclosure not accessible to visitors. This situation, which was unfamiliar to the meerkats, was apparently considered threatening, causing alarm calls to be uttered. Since no birds of prey or other vocalizing predators are kept near the meerkat enclosure either, we assume that both the graded structure of alarm calls and a natural response to acoustic stimuli are preserved in other zoos, as well. This could be confirmed in further studies.

## 5. Conclusions

In this paper, we demonstrated that captive meerkats show biologically relevant and selective responses to potential acoustic threats before rapidly returning to baseline activity. In addition, playback experiments indicated that the necessary response to alarm calls is preserved, as well. Feature and cluster analysis further revealed the preservation of the graded structure of emitted alarm calls in the zoo. By demonstrating a fully preserved natural response to acoustic stimuli and the graded structure in alarm calls, further bioacoustics studies regarding natural behaviors and needs can also be conducted in zoos. Furthermore, zoos can benefit from the knowledge gained from this study and draw conclusions about adverse environmental conditions for their meerkat husbandry by means of appropriate data recording. In this way, husbandry conditions and thus animal welfare can be improved.

## Figures and Tables

**Table 1:** Overview of the different experiments. Stimuli marked with an * were additionally combined with a visual stimulus. Since dogs are allowed in the zoo, they are considered local.

Part of the Study	Stimulus	Alarm Call	Local to the Zoo	Iterations
Behavior maintenance and call acquisition experiments	Common buzzard * (*Buteo buteo*)	Aerial	Yes	6/6 *
	Red kite (*Milvus milvus*)	Aerial	Yes	7
	Robin (*Erithacus rubecula*)		Yes	7
	Blue tit (*Cyanistes caeruleus*)		Yes	9
	Cuckoo (*Cuculus canorus*)		Yes	7
	Common raven (*Corvus corax*)	Aerial	Yes	8
	Jackal buzzard (*Buteo rufofuscus*)	Aerial	No	6
	Common jackal (*Canis aureus*)	Terrestrial	No	9
	domestic dog (Weimaraner)	Terrestrial	(Yes)	3
Playback experiments	Meerkat alarm call (*Suricata suricatta*)	Aerial		10
	Robin (*Erithacus rubecula*)		Yes	10
Feature and cluster analysis	Common buzzard * (*Buteo buteo*)	Aerial and Terrestrial		6 *

**Table 2:** Used ethogram of meerkat behavior.

Behavior Category	Behavior
environment-directed behavior	guard
	observe
self- and intraspecies- directed behavior	foraging
	rest
	eat
	others
flight	flight
walk	walk
n.v.	not visible

**Table 3:** Features selected via the LASSO algorithm per alarm call type. Feature abbreviation: Duration, duration of the vocalization; F0, fundamental frequency; F1, first formant; ΔF0-F1, delta between F0 and F1; Harmonic ratio, describes how harmonic or noisy a signal is; Spectral flatness, quantify how tone-like or noise-like a sound is; DFA1, frequency at which the energy reaches the first quartile; DFA2, frequency at which the energy reaches the second quartile; DFA1maloc, location of the maximum frequency in the first quartile; DFA2maloc, location of the maximum frequency in the second quartile. A list of all used features can be found in Table S1.

Aerial Alarm Call	Terrestrial Alarm Call
Duration	Duration
F0	F0
F1	ΔF0-F1
Harmonic ratio	Harmonic ratio
Spectral flatness	DFA1
	DFA2
	DFA1maloc
	DFA2maloc

## Data Availability

The data presented in this study are available on request from the corresponding author. The data are not publicly available due to the fact that the audio recordings may contain voices of zoo visitors or zoo staff whose consent for publication we do not have.

## Supplementary Materials

The following are available online at https://www.mdpi.com/article/10.3390/ani11113064/s1, Table S1: List of acoustic features extracted for each vocalization. If applicable, the corresponding MATLAB Toolbox is listed in the References column followed by the command used (in brackets), Table S2: Used ethogram of meerkat behavior with behavior definition.
