# Supplementary material for: Discrimination of Acoustic Stimuli and Maintenance of Graded Alarm Call Structure in Captive Meerkats
Source: Animals (Basel). 2021 Oct 27;11(11):3064. doi: 10.3390/ani11113064 (PMC8614505; doi:10.3390/ani11113064)
Supplement: Supplementary file 1 [file animals-11-03064-s001.zip › animals-1384943-supplementary.pdf]

Table S1. List of acoustic features extracted for each vocalization. If applicable, the corresponding MATLAB Toolbox is listed in the References column followed by the command used (in brackets).

Features	Definition of Features	Reference
Delta fund	Median value of the difference between adjacent values for F0 per time window.	
Dominant f	Frequency with highest Amplitude in the spectrum	
Min f	Lower bound of the 99% occupied bandwidth	MATLAB Signal Processing Toolbox ("obw")
Max f	upper bound of the 99% occupied bandwidth	MATLAB Signal Processing Toolbox ("obw")
Bandwidth	The 99% occupied bandwidth	MATLAB Signal Processing Toolbox ("obw")
Duration	Time between onset and offset of a vocalization in seconds.	
DFA1	frequency at which the energy reaches the first quartile	[1]
DFA2	frequency at which the energy reaches the second quartile	[1]
DFA3	frequency at which the energy reaches the third quartile	[1]
DFA1maloc	Frequency with highest amplitude in 1st Quartile	
DFA2maloc	Frequency with highest amplitude in 2nd Quartile	
DFA3maloc	Frequency with highest amplitude in 3rd Quartile	
DFA4maloc	Frequency with highest amplitude in 4th Quartile	
F0	Median frequency of the 1st Formant-like structure (Hz)	LPC with 205 coefficients via MATLAB Signal Processing Toolbox ("lpc")
F1	Median frequency of the 2nd Formant-like structure	LPC with 205 coefficients
F2	Median frequency of the 3rd Formant-like structure	LPC with 205 coefficients
BW F1	Median Bandwidth of F1	MATLAB ("findpeaks")
BW F2	Median Bandwidth of F2	MATLAB ("findpeaks")
BW F3	Median Bandwidth of F3	MATLAB ("findpeaks")
Δ F0-F1	Difference of F1 and F2	
Δ F1-F2	Difference of F2 and F3	
Harmonic Ratio	The harmonic ratio is returned with values in the range 0 to 1. A value of 0 represents low harmonicity, and a value of 1 represents high harmonicity.	MATLAB Audio Toolbox ("harmonicRatio")
Spectral Flatness	Measures how noisy a signal is. The higher the value, the noisier the signal.	MATLAB Audio Toolbox ("spectralFlatness")

[1] Schrader; Lars; Hammerschmidt; Kurt. Computer-aided analysis of acoustic parameters in animal vocalisations: A multi-parametric approach. Bioacoustics 1997, 7, 247–265.

Table S2. Used ethogram of meerkat behavior with behavior definition.

Behavior Category	Behavior	Definition
environment-directed behavior	guard	The animal maintains an upright posture and its head moves frequently from left to right, surveying the surrounding area. It stands on the toes of its hind legs supported by its tail resting on the ground.
	observe	The animals head moves frequently from left to right, surveying the surrounding area, standing on all four feet or in a sitting position.
self- and intraspecies-directed behavior	foraging	Individuals digging or foraging for food.
	rest	The animal laying on the ground while all four feet contact the ground.
	eat	Individuals eating food.
	others	Individuals do not survey the area. They are engaging in comforting behavior such as playing or grooming.
flight	flight	Individuals are running away, into the burrow or in-door enclosure through the enclosure wall.
walk	walk	Individuals are moving from one point to another.
n.v.	not visible	Individuals are not visible. They can either be in the caves or inner enclosure, or may be hiding behind rocks or shrubbery.
